# Case Report: Possible C3 nephritic factor–driven complement-mediated severe hemolytic anemia and acute kidney injury in a child with *Bordetella parapertussis* infection

**DOI:** 10.3389/fimmu.2025.1715464

**Published:** 2026-01-02

**Authors:** Steffen Ullitz Thorsen, Anne Todsen Hansen, Hans Jakob Hartling, Hanne Vibeke Marquart, Line Borgwardt, Mira Marie Laustsen, Lillemor Skattum, Alex Christian Yde Nielsen, Line Thousig Sehested, Hanne Nørgaard, Morten Hanefeld Dziegiel, Ida Maria Schmidt

**Affiliations:** 1 Department of Clinical Immunology, Rigshospitalet, University of Copenhagen, Copenhagen, Denmark; 2 Institute of Clinical Medicine, Faculty of Medicine, University of Copenhagen, Copenhagen, Denmark; 3 Center for Genomic Medicine, Rigshospitalet, University of Copenhagen, Copenhagen, Denmark; 4 Clinical Immunology and Transfusion Medicine, Lund University Hospital, Lund, Sweden; 5 Department of Laboratory Medicine, Section Microbiology, Immunology and Glycobiology (MIG), Lund University, Lund, Sweden; 6 Department of Clinical Microbiology, Rigshospitalet, University of Copenhagen, Copenhagen, Denmark; 7 Department of Pediatrics, Rigshospitalet, University of Copenhagen, Copenhagen, Denmark

**Keywords:** pediatrics, hemolysis, alternative complement pathway, atypical hemolytic syndrome, C3 nephritic factor, acute kidney injury

## Abstract

Overactivation of the complement system can cause life-threatening intravascular hemolysis, acute kidney injury (AKI), and multi-organ failure. Expanding the spectrum of rarer triggers of complement dysregulation that may cause severe hemolysis is essential for timely diagnosis and treatment. We report the case of a three-year-old boy admitted with macroscopic hematuria and jaundice. The patient tested positive for *Bordetella parapertussis*. Atypical hemolytic uremic syndrome (aHUS) was initially suspected due to decreasing hemoglobin, platelet count, and worsening of AKI, and empirical treatment with eculizumab (ECZ) was initiated. No red blood cell (RBC) autoantibodies were detected by standard serology methods or extended flow cytometry including antibody fixation at both room temperature and 4°C, no underlying hematological disorder was found, and genetic screening revealed no pathogenic variants associated with aHUS. However, the patient was strongly positive for C3 nephritic factor (C3NeF), an autoantibody stabilizing the C3 convertase (C3bBb) of the alternative complement pathway. C3NeF is typically linked to C3 glomerulopathy, but in this case appears to have triggered severe complement overdrive and extra- and intravascular hemolysis (bystander hemolysis) during an infection (two-hit immunopathology). Concomitantly, *B. parapertussis* is known to bind complement factor H, a key regulator of the alternative pathway, creating a “perfect storm” of dysregulation. Following infection control and complement blockade with ECZ, C3d deposition on RBCs declined, renal function recovered, and no clinical relapse has been observed 2 years and 6 months after discharge. To our knowledge, this is the first reported case of a possible C3NeF-driven complement-mediated severe hemolysis with associated AKI due to free hemoglobin toxicity during a severe infection.

## Introduction

The complement system is an important defense mechanism against pathogens; however, in certain pathologies, the system also attacks human host cells, such as red blood cells (RBCs) (1, 2). RBCs seem particularly sensitive to dysregulation of the complement system, which is not surprising as RBCs are continuously exposed to activated complement components. Premature—and sometimes fulminant—destruction of RBCs can lead to life-threatening anemia and organ failure. This may result from either an intrinsic defect in the complement system, such as genetic mutations in complement regulators, or from extrinsic triggers, including specific infections or autoantibodies that disrupt the balance between complement activation and regulation. A well-known example is autoantibodies against complement factor H (anti-CFH), which lead to excessive activation of the alternative complement pathway (1).

When a child presents with infection, intravascular hemolytic anemia, and kidney failure, most pediatric nephrologists immediately consider hemolytic uremic syndrome (HUS) as a likely diagnosis that requires further evaluation. HUS is a thrombotic microangiopathy (TMA) characterized by complement-mediated and mechanical intravascular hemolysis resulting in varying degrees of RBC fragments (schistocytes), thrombocytopenia, and AKI (3). HUS is usually divided into three categories: i) Typical HUS caused by Shiga toxin-producing *Escherichia coli* (STEC-HUS) infection; ii) atypical/complement-mediated HUS (aHUS) usually caused by dysregulated complement activation; and iii) secondary HUS with coexisting disease, e.g., infection with *Streptococcus pneumonia* or *Influenza* virus. Diagnostic clarity including testing for pathogenic variants in genes associated with defects in the complement system is time-consuming and fails to identify underlying abnormalities in the system in 40% of patients (4). Therefore, treatment is often started empirically. Eculizumab (ECZ), is a monoclonal therapeutic antibody that targets complement factor 5 (C5) and thereby inhibits the formation of the terminal complement complex (TCC), has been a game-changer in the treatment of aHUS and complement-driven intravascular hemolysis, but comes with high economical cost and an increased infection risk, especially with *Neisseria* spp. (2). Rare differential diagnoses that clinically mimic aHUS must be considered to ensure an accurate diagnosis, appropriate treatment, and follow-up. C3-nephritic factor (C3NeF) is an autoantibody generated against neoepitopes in the C3 convertase of the alternative complement pathway (C3bBb). By stabilizing the convertase and prolonging its natural degradation, “pathogenic” C3NeF can lead to excessive complement activation and C3 hypocomplementemia in non-infective states, which may increase the risk of bacterial infections (5, 6). Typically C3NeF is linked to C3 glomerulopathy, but can also be found in a small number of healthy subjects (6). The literature on “silent” C3NeF that may only be “pathogenic” during considerable alternative complement pathway activation, e.g., severe systemic bacterial infections (the two-hit immunopathology hypothesis), is scarce.

We present a novel and puzzling case with severe intravascular hemolysis, mild thrombocytopenia, and AKI with preserved diuresis during a *Bordetella parapertussis* infection, which initially was diagnosed and treated as HUS. However, the boy tested strongly positive for C3NeF. As the triggering event—an infection—came under control, the overactivation of the alternative complement pathway subsided as did the severe bystander hemolysis (non-discriminatory complement-deposition on RBCs) (7, 8), which was also accompanied by a considerable decrease in C3NeF levels. Moreover, we present a flow cytometric method for accurately monitoring complement-mediated destruction of RBCs. This approach may serve as a valuable tool for pediatric nephrologists and immunologists in assessing alternative complement pathway activation—specifically, ongoing C3d deposition on RBCs during treatment with ECZ or other C5 inhibitors.

## Case presentation

A three-year-old boy presented with a one-week history of on-off fever, fatigue, and upper airway symptoms, e.g., coughing and tachypnea, that progressively got worse. Macroscopic hematuria and icterus of skin and sclera caused admission to our acute pediatric in-patient clinic. There was no history of diarrhea.

The patient had no prior medical history, no recent foreign travel, did not take any conventional or alternative medicine, and followed the Danish vaccination program which includes vaccination against *Bordetella pertussis*. He had no notable family history of either hematological, immunological, or kidney diseases; he is 3/4 ethnic Danish and 1/4 Kenyan. Furthermore, he had a normal metabolic screen at birth and had developed appropriately for his age.

**Figure 1:**
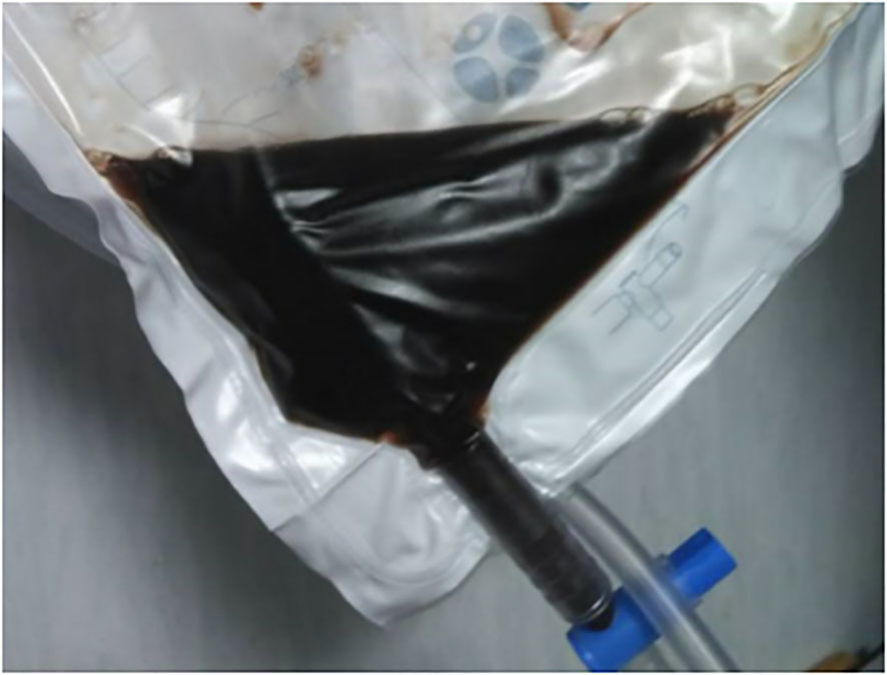
*Black water urine* developed the day after admission and following red blood cell transfusions.

On examination, he presented with septicemia-like distress. Laboratory results showed mild anemia (6.1 mmol/L), thrombocytosis (570x10^9^ cells/L), leukocytosis (34.1x10^9^ cells/L with neutrophil dominance (23.9x10^9^ cells/L), C-reactive protein (CRP) (170 mg/L), hyperbilirubinemia (98 μmol/L), increased creatinine (117 μmol/L) and alanine transaminase (95 U/L) (**Table 1**). Temperature was 39.4 °C, macroscopic hematuria, which during the first 24 hours of admission turned black (*“black water urine”*, **Figure 1**), ferritin >2000, plasma free hemoglobin 19 µmol/L (reference: 0–3 µmol/L) (**Table 1**), schistocytes 2% (considered normal).

**Table 1:** Overview of routine and specialized laboratory results, blood transfusions, and administration of antibiotics and eculizumab.

Day after admission	1	2	3	4	5	6	7	8	9	10	11	12	13	14	15	16	17	26	39	180	310	859
Hematological and inflammation markers																						
Hemoglobin (ref.: 6.5-8.9 mmol/L)	6.1	5.1	4.1	3.9	6.0	4.9	4.7	4.4	4.3	4.0	4.2	4.0	4.1	4.1	3.5	4.0	4.4	5.6	6.7	7.0	7.6	7.4
Lactate dehydrogenase (ref.: 155–450 U/L)	–			2430	2384	1560	1350	1310	1090	997	843	773	686	656			430	431				
Bilirubin (ref.: 5–25 umol/L)	98	87	58	20	49	<20	18	8	7	7	6	6	6				3		<20	5	<20	
Plasma free hemoglobin (ref.: 0-3 µmol/L)		19	23*	3	98*	5		3	<3	3		<3	<3	<3	<3	<3	<3					
Reticulocytes (ref.: 29-83 × 10&^9^/L)	41	32	43	49	90	104				172				116				156				
Platelet count (165-435 × 10&^9^/L)	570	319	320	163	91	108	157	213	278	340	380	440	425	410	410	456	594	436	366	315	359	389
C-reactive protein (ref.: < 10 mg/L)	170	155	136	73	59	51	36	31	27	20	13	7	5	5	4	2	1	3				<1
Kidney function																						
Creatinine (ref.: 23-37 µmol/L)	117	152	254	324	349	383	377	350	285	203	109	62	56	56	48	43	33	28	25	27	27	35
Urine output (ml/kg/t)		1.6	3.4	2.6	3.8	3.6	4.0	4.3	5.7	5.2	4.9	5.3	3.8	3.8	2.7	2.4	2.9					
Treatments																						
Blood transfusion			X	X																		
Antibiotics: Meropenem Azithromycin Amoxicillin*** Pip/tazo**	X	X	X X	X X	X X	X X	X X	X	X	X	X	X	X X	X	X	X	X		X			
Eculizumab					X							X										
Complement system factors																						
C3 nephritic factor (ref: <15%)		91%																39%			20%	41%
C4 nephritic factor		Neg.																Neg.				
C3 (ref.: 0.76-1.77 g/L)		0.44																1.11				1.02
C4 (ref: 0.13-0.39 g/L))		0.011																0.22				
Factor H (%) (69-154%)		58																				
TCC/C5b-C9 (ref.: < 190 ng/mL)		793				83						104	75									

Note that reference intervals are based on the patient’s age at admission; however, these values change only slightly over the course of the follow-up period. Of note, the complement assays does not have age-specific reference intervals.

*Measured after red blood cell transfusion.

**Piperacillin and Tazobactam due to fever. Stopped when cultures came back negative.

***Amoxicillin was continued until almost 3 months after Eculizumab, when complement screening was normalized.

, LDH could not be estimated due to hemolysis in the sample.

The patient tested positive for *Bordetella parapertussis* in a BioFire RP2.1 test using the respiratory panel (https://www.biofiredx.com/%20products/the-filmarray-%20panels/filmarrayrp/) (9)—this finding was confirmed with a specific PCR-test using the InGenius platform. The patient tested negative for a panel of upper-airway viruses, hepatitis A and B, HIV, CMV, EBV, Parvo B19, and current COVID-19 infection. Furthermore, feces samples were negative for common viral and bacterial pathogens including STEC (O26, 103, 111, 145, 157, 177). Lastly, the patient was also serologically tested negative for *Bartonella henselae* and *quintana*.

**Figure 2:**
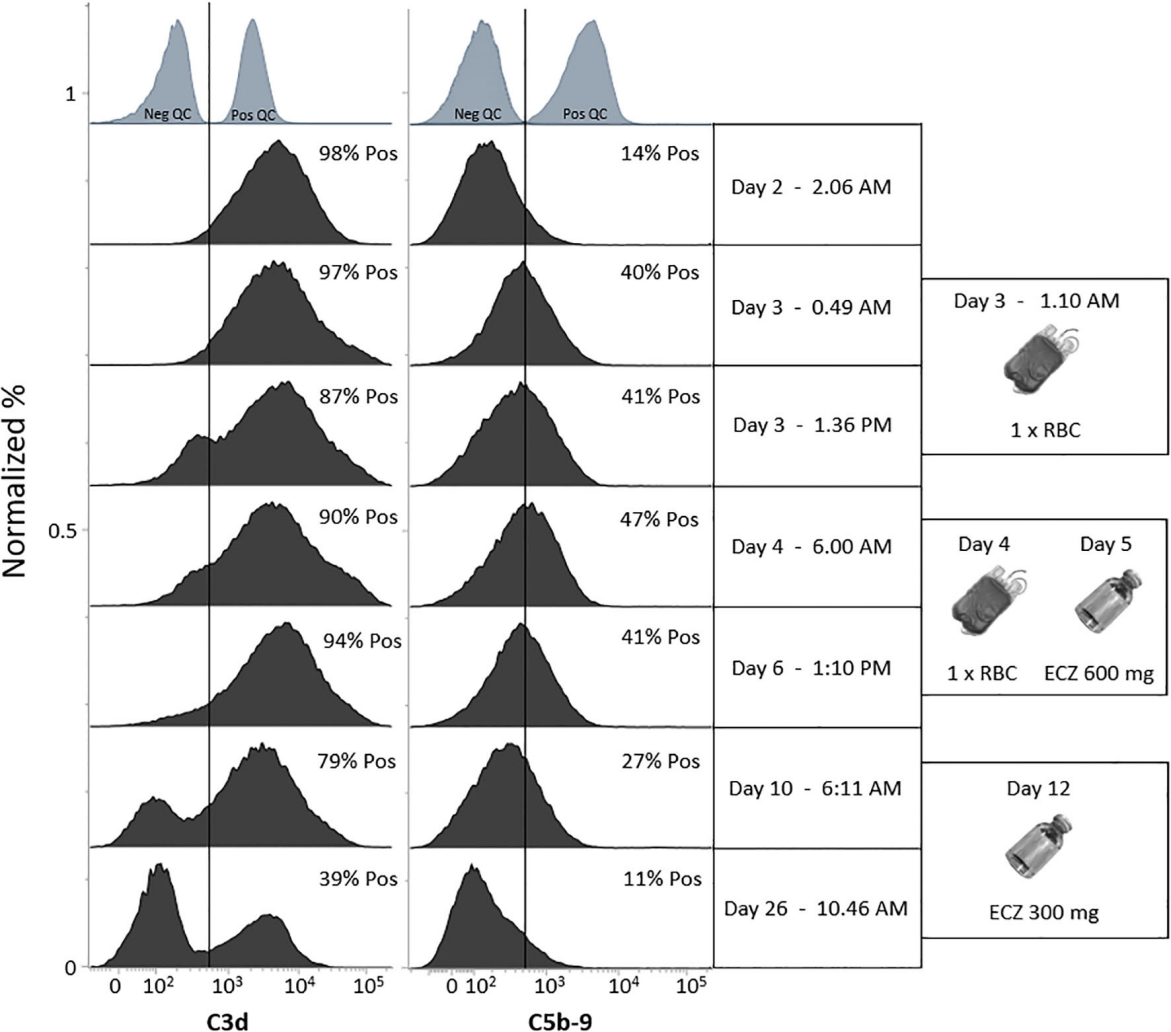
A series of stacked histograms showing the deposition of complement components C3d and C5b-9 on red blood cells (RBCs) over the first 25 days following admission. A gate based on negative controls was applied to each stack of histograms, and the percentage of complement-positive RBCs was determined individually. In most histograms, a shift of the entire RBC population was observed, indicating that all RBCs were coated with complement. Following the RBC transfusion on day 3, the donor RBCs were rapidly coated with C3d, reflecting an overactivation of the alternative complement pathway. By day 10, a distinct C3d-negative RBC population emerged. This population continued to increase and became dominant by day 26, suggesting that the patient’s newly produced RBCs were no longer coated with complement at the C3 level. This finding indicates a reduction or normalization of alternative complement pathway activity.

Immunohematological testing revealed no evidence of RBC autoantibodies, as assessed by standard serology and extended flow cytometry under both unfixed and fixed conditions at room temperature and 4 °C, designed to optimize detection of low-affinity autoantibodies across a broad thermal range and across IgA, IgG, and IgM isotypes (*method paper in preparation*). However, the patient tested strongly positive for C3d using gel card analysis (DC Screening II, DiaMed GmbH, Ref. 004831) and flow cytometry (**Figure 2**) (10). Suspicion of paroxysmal cold hemoglobinuria (PCH) was ruled out after a negative Donath-Landsteiner (D-L) test (11). A broad autoimmune screen was negative including autoantibodies associated with immune complex formation. Slightly reduced levels of ADAMTS13-protein were measured (0.58 kIU/L: ref: 0.61-1.31 kIU/L) (12). Furthermore, the patient was found heterozygote for α-thalassemia (-α,αα) — a clinically silent form of thalassemia (13). Tests for other hemoglobinopathies, including sickle cell anemia, were negative. We also found normal glucose-6-P-dehydrogenase activity in RBC and a normal paroxysmal nocturnal hemoglobinuria (PNH) clone panel (CD157 and FLAER negative neutrophils and monocytes). No inborn errors of immunity (IEI) were detected using immunophenotyping, which was performed with a custom-designed 10-color flow cytometry prefabricated freeze-dried antibody panel (DuraClone, Beckman Coulter, Brea, CA), specifically developed for evaluating leukocyte subsets in patients with IEI and secondary immunodeficiencies (14).

**Figure 3:**
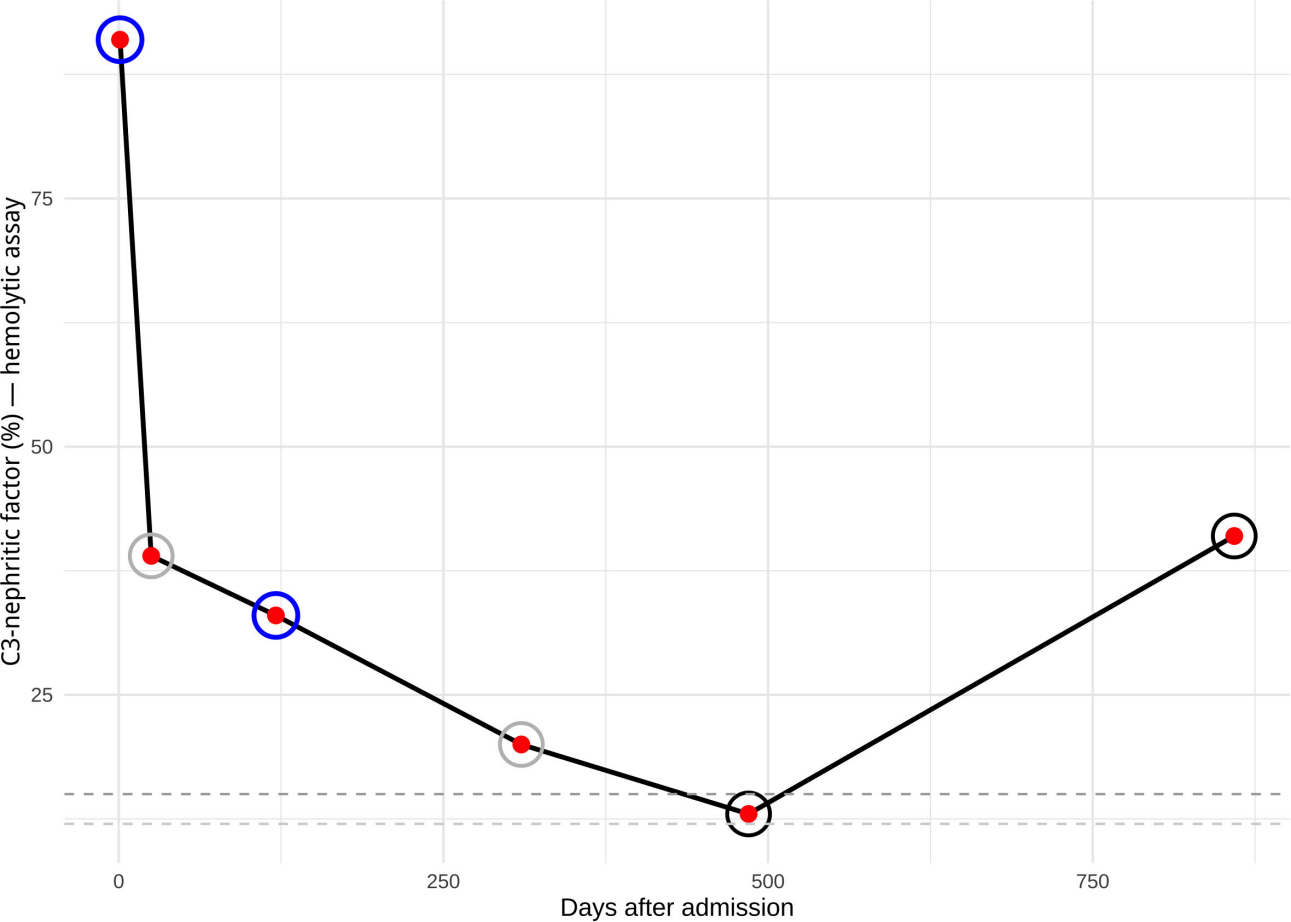
Levels of C3-nephritic factor (C3NeF) factor using the hemolytic assay (%) from initial admission and follow-up in the pediatric nephrological outpatient clinic. During the observation period, the hemolytic assay used to measure C3NeF changed, making direct comparison between early and later measurements challenging. Nonetheless, the graph is intended to illustrate the overall trend in C3NeF levels over time, which reflected the patient’s clinical remission. The reference threshold for normal levels also changed slightly between assays—from 15% (indicated by the dark grey dashed line) to 12% (light grey dashed line). Note: Rings around the time points indicate results from the ELISA assay for C3NeF: dark blue denotes a positive result, black a negative result, and grey indicates that no ELISA measurement was performed.

Initially, the patient had both C3 (0.44 g/L) and C4 (0.011 g/L) hypocomplementemia and increased total complement complex (TCC/C5b-C9) (793 ng/mL); normal levels of factor B, factor I, and slightly reduced factor H levels (58%, reference interval 69–154%). No anti-CFH were detected. The patient was strongly positive for C3NeF in a functional hemolytic assay based on sheep RBC (15) as well as an ELISA assay (16) (**Figure 3**). C4 nephritic factor (C4NeF) was analyzed *post hoc* on the same days as C3NeF using a modified version of the assay described by Blom et al. (2017) (17), and the results were negative (**Table 1**)—so no sign of NeF double positivity (18). Whole genome sequencing (WGS) was performed using in silico in-house gene panel including 52 genes associated with aHUS (**Supplementary Table 1**) and three important risk factors for aHUS including two single nucleotide polymorphisms haplotype blocks: the CFH-H3 haplotype and the MCPggaac haplotype and a well-known *CFHR3/CFHR1* deletion (19–21). No pathogenic variants in these genes were identified, neither in the haplotypes nor in the deletion.

During the first days following hospital admission, the patient’s condition progressed, leading to a worsening of the three hallmark symptoms of HUS: AKI (creatinine 344 μmol/L), mild thrombocytopenia [nadir day 5 after admission (91x10 (9) cells/L)], and anemia (3.9 mmol/L), however schistocytes were < 2%. On the third day after hospital admission, the patient was started on Azithromycin treatment (5 mg/kg/daily for 5 days) for his *Bordetella parapertussis* infection leading to disease control around day 6 after admission. On the fifth day after hospital admission, ECZ (600 mg once) was initiated due to suspected aHUS, prior to the result of WGS and the extended complement screen including C3NeF testing—a functional complement screen analysis at the day of ECZ initiation showed marked suppression in the classical and alternative complement pathways (data not shown) (22), which is in line with his initial C3 hypocomplementemia. A second dose of ECZ (300 mg once) was administered seven days later.

Two RBC transfusions were administered on the second and third day after admission, following negative screening tests for anti-RBC antibodies. After both transfusions, the patient experienced acute hemolytic transfusion reactions (HTRs) with a rise in fever, shivers, and black urine. Plasma-free hemoglobin rose to very high levels accompanied by increased creatinine levels (maximum at 383 μmol/L two days after the second RBC transfusion (day 6 after admission) (**Table 1**)). The clinical symptoms associated with the RBC transfusion complications subsided within a few hours, and the urine discoloration returned to normal.

The patient’s clinical condition and clinical laboratory tests normalized, i.e., resolution of anemia, mild thrombocytopenia, and AKI (**Table 1**). He was never anuric, thus no hemodialysis or plasmapheresis was performed. The patient never received any systemic immunosuppressive therapy (e.g., glucocorticoids) during either the acute phase or the remission period. No clinical relapse has been seen during the 2 years and 6 months after discharge. He remained on Amoxicillin for nearly three months post-ECZ treatment as a precaution, until his functional complement screen analysis returned to normal. Initially, C3NeF levels decreased significantly (**Figure 3**). C3NeF remained only marginally detectable in the hemolytic assay and was negative in the ELISA test. However, two years and five months after his initial admission, an increase in C3NeF was observed in the hemolytic assay (no parental anamnestic information indicating any significant infections prior to testing), though this was not accompanied by signs of increased alternative complement pathway activation, as C3 levels remained within the normal range (**Figure 3**, **Table 1**). The patient has remained asymptomatic; however, he has not since experienced another severe bacterial or viral infection comparable to the one described in this case report.

## Discussion

We report an atypical disease course initially resembling HUS, which was later interpreted as a severe complement-mediated hemolysis with C3 hypocomplementemia, triggered by a severe bacterial infection (*Bordetella parapertussis*) and exacerbated by C3NeF (the two-hit immunopathology hypothesis)—a novel sort of bystander hemolysis (5, 7). The complement-mediated hemolysis led to AKI due to high levels of free hemoglobin, which was worsened by RBC transfusions (23, 24). Noteworthy, increased levels of heme, a breakdown product of hemoglobin, may in a dose-dependent manner inhibit factor I-mediated degradation of C3b, thereby further fueling ongoing hemolysis (25). The fact that his schistocyte count was normal, and that he presented with anemia accompanied by thrombocytosis, may have initially guided us away from considering HUS as the provisional diagnosis. This case report illustrates the importance of multidisciplinary teamwork regarding the diagnosis, treatment, monitoring, and re-evaluation of diagnosis in the scenario of severe overdrive of the alternative complement pathway, initial thrombocytosis/later mild thrombocytopenia, extra- and intravascular hemolytic anemia, and AKI.

Our patient presented initially with severe complement-mediated hemolysis (extra- and intravascular) with all RBCs coated with C3d as demonstrated by flow cytometry. No evidence of autoimmune hemolytic anemia (AIHA) was found, including neither warm- nor cold-reactive IgG, IgA, or IgM autoantibodies, PCH (a negative D-L test and negative in RBC flow cytometry with fixation of possible cold-reacting RBC autoantibodies at 4 °C), PNH (2) or RBC T-activation (26) were observed. In summary, this extended examination significantly reduces the risk of overlooking low-affinity RBC-targeted autoantibodies with varying thermal amplitudes. No signs of mechanical hemolysis (TMA) were present i.e., schistocyte count were within normal range. The strongly positive C3NeF test—the results were first available three weeks after admission—was initially overlooked amid numerous clinical findings and was not fully considered as a possible primary driver of alternative complement pathway overactivation in concert with a severe bacterial infection. We observed a marked decrease in C3NeF levels from day 2 to day 26 after admission (from 91% to 39%), which may be attributed to the initiation of ECZ (27). Notably, similarly rapid declines in other complement-related autoantibodies, such as anti-CFH, have been reported following the start of immunosuppressive therapy (28), which is probably due to a marked reduction in complement activation and inflammation. In addition, fluctuations in C3NeF levels have previously been reported and do not necessarily correlate with clinical parameters (18), as reported in this case and illustrated in **Figure 3**. Further, the initial C4 hypocomplementemia may be explained by activation of the classical (and to some extent lectin) pathway, due to presence of antibodies against *Bordetella parapertussis*, consistent with symptoms of an airway infection one week prior to admission (29–31). Notably, both C3 and C4 levels had normalized by day 26 after admission (**Table 1**).

ECZ therapy was initiated early based on a tentative aHUS diagnosis, alongside targeted antibiotics for *Bordetella parapertussis*. However, ECZ (or emerging C3 inhibitors) may still represent appropriate first-line treatment in C3NeF-driven hemolysis, as it halts hemolysis and prevents free-hemoglobin–induced nephrotoxicity. Nevertheless, this approach does not target C3NeF production itself, which may require immunosuppressive therapy such as corticosteroids and potentially mycophenolate mofetil (MMF) (5, 6) or acute removal using therapeutic plasmapheresis. Regarding the latter, timely processing of C3NeF testing is essential for it to inform appropriate clinical decision-making.

Virulence factors of *Bordetella parapertussis* might have aggravated the complement-mediated hemolysis and thereby creating “a perfect storm”. Importantly, Almdahl et al. showed *Bordetella pertussis and parapertussis* utilizes CFH binding as a mechanism to inhibit complement activation and enhance immune evasion (32). However, the virulence factors of *Bordetella parapertussis* are considered less effective at immune evasion than those of *Bordetella pertussis*. As shown in **Table 1**, circulating CFH concentrations were slightly reduced, which may reflect increased microbial surface binding and/or enhanced utilization due to overactivation of the alternative complement pathway. Obviously decreased circulating CFH levels may have reduced the RBCs defenses against complement-mediated destruction. It remains unclear whether the patient was C3NeF-positive prior to the infection or if the infection triggered C3NeF production.

The pathology of aHUS has been extensively reviewed (3), and the common belief is that hemolysis in aHUS is not mediated solely by complement but predominantly as a result of mechanical hemolysis. However, very recent data obtained in an *in vitro* model of aHUS showed that hemolysis can be a direct result of the complement system alternative pathway activity as well (33). However, considering a strong positive C3NeF, the absence of schistocytes, and no history of severe thrombocytopenia, the *a priori* risk of aHUS may be considered low. Moreover, we did not identify pathogenic variants in genes included in the aHUS gene panel, however this does not rule out a genetic cause of aHUS. Furthermore, Arjona et al. have shown that different genetic risk factors contribute to the penetrance of aHUS and suggests that penetrance score values have an inverse correlation with the intensity of environmental triggers required for disease development (20). We investigated the same haplotypes as Arjona et al., specifically the CFH-H3 and MCPggaac haplotypes, and additionally analyzed the well-documented *CFHR3/CFHR1* deletion. However, no genetic risk factors were detected.

We propose that in cases of severe intravascular hemolysis and uncertainty regarding diagnosis and empirically use of complement inhibition i.e., ECZ, monitoring of C3d deposition on RBC by flow cytometry, may be clinically valuable for in-depth evaluation of alternative complement pathway overdrive cessation, and can thereby guide further treatment strategy (**Figure 2**). By using flow cytometry in this patient we were able to identify the advent of a novel population of RBC (freshly produced from the patient’s own bone marrow) not coated with C3d during ECZ treatment indicating a halt in the alternative complement pathway overdrive—of notice ECZs therapeutic target (C5) is downstream of the C3 convertase, so even though C5b-C9 generation is inhibited by ECZ and hereby intravascular hemolysis C3b/d depositing is still possible and may lead to non-neglectable extravascular hemolysis as seen in patients with PNH (34, 35). Furthermore, by using alternative methods to monitoring complement activation such as circulating C3d and TCC/C5b-9 (**Table 1**) one might see a decrease in levels, but not get a clear idea of actual deposition on RBCs. However, circulating C5b-9 is a cheaper and easier way to monitor the effects of ECZ treatment alongside functional testing of inhibition of all three complement pathways (22). When using flow cytometry to monitor C5b-9 deposit on RBCs during ECZ therapy we recommend to examine suspicious negative results with an anti-mouse antibody test (the heavy-chain in ECZ is of mouse origin) to unmask a false negative test, due to competitive binding of anti-C5b–9 and ECZ—the latter has been shown to bind to soluble C5b (36).

Notably, it is shown in **Figure 2** how quickly donor RBCs from transfusion are coated with C3d/C5b-9 in our patient after transfusion greatly minimizing donor RBC survival and thereby the intended treatment effect, while further inducing kidney damage due to free hemoglobin (23). This prompts us to question whether RBC transfusions should be avoided, if feasible, in the presence of complement overactivity when PNH is not suspected—similar to the caution exercised with platelet transfusions in both HUS and aHUS. In our case, increased kidney damage became apparent following RBC transfusions. If RBC transfusions are indicated, complement inhibition therapy should be initiated beforehand. Alternatively, a slow RBC transfusion is advisable to monitor for the development of acute HTRs. Lastly, although the donor RBC units were less than 10 days old, it is likely that the oldest RBCs within the products experienced the most extensive complement-mediated destruction, due to age-related decline in membrane-bound complement regulatory proteins (37, 38).

Lastly, several limitations of this case report should be acknowledged: (i) C3NeF as a primary driver of bystander hemolysis in acute severe infection remains an exclusion diagnosis, as direct causality cannot yet be proven; (ii) C3NeF levels prior to disease onset were unavailable, so it remains unclear whether the infection triggered autoantibody generation or simply acted as a sufficient inflammatory trigger in a patient with pre-existing C3NeF (the two-hit immunopathology hypothesis); and (iii) we did not perform staining for classical complement components (e.g., C4d) on RBC membranes, which could have helped exclude undetected RBC-directed autoantibodies or immune complexes capable of interacting with RBCs.

## Conclusion

We present what appears to be the first reported possible case of C3NeF-associated severe intravascular hemolysis in the absence of RBC autoantibodies or mechanical hemolysis. The patient—a three-year-old boy with a severe *Bordetella parapertussis* infection—was initially treated for HUS based on the classic triad of anemia, thrombocytopenia, and AKI. However, the absence of schistocytes and presence of a strong positive C3NeF test should have prompted reconsideration of the diagnosis in favor of pure complement-mediated hemolysis driven by alternative complement pathway overactivation during a severe bacterial infection (bystander hemolysis). That may have been worsened by *Bordetella parapertussis* ability to bind CFH. In this context, intravascular hemolysis and the resulting free hemoglobin contributed substantially to the AKI. Notably, RBC transfusions provided no clinical benefit and aggravated the AKI.

Finally, we propose, if possible, to incorporate real-time monitoring of complement deposition on RBCs via flow cytometry throughout the hospital stay when severe complement-mediated hemolysis is suspected. This approach may support early diagnosis and guide both treatment decisions and assessment of therapeutic response.

## Data Availability

The original contributions presented in the study are included in the article/**Supplementary Material**. Further inquiries can be directed to the corresponding author.
