# Supplementary material for: Case Report: Possible C3 nephritic factor–driven complement-mediated severe hemolytic anemia and acute kidney injury in a child with Bordetella parapertussis infection
Source: Front Immunol. 2026 Jan 2;16:1715464. doi: 10.3389/fimmu.2025.1715464 (PMC12808469; doi:10.3389/fimmu.2025.1715464)
Supplement: Supplementary file 1 [file Table1.docx]

**Supplementary Materials**

**Supplementary Table 1**

List of in silico in-house gene panel including 52 genes associated with aHUS

*ARMS2, CFHR1, CR2, CFHR3, C6, CFI, C7, CD46, C3AR1, C8A, CD59, C1S, C1QB, C3, COLEC12, C2, CFH, CFHR4, C8G, MMACHC, COLEC10, DGKE, THBD, PTX3, C1QC, C1INH, C4, C8B, C9, CR1, CFHR5, C5, C1QA, C4BPA, C4B, C1R, CFD, FCN2, MASP1, CFB, C5AR1, FCN1, CD55, MASP2, MBL2, COLEC11, FCN3, VTN, ADAMTS13, C4BPB, HTRA1, CFHR2.*
